# An investigation on the presence of *Chlamydiaceae *in Swedish dogs

**DOI:** 10.1186/1751-0147-52-63

**Published:** 2010-11-16

**Authors:** Bodil Ström Holst, Sofia Hanås, Göran Bölske, Catharina Linde Forsberg

**Affiliations:** 1 National Veterinary Institute (SVA), Uppsala, Sweden; 2 Swedish University of Agricultural Sciences (SLU), Faculty of Veterinary Medicine and Animal Health, Department of Clinical Sciences, Uppsala, Sweden; 3 Animal Clinic Djurdoktorn, Västerås, Sweden

## Abstract

**Background:**

Bacteria belonging to the family *Chlamydiaceae *cause a broad spectrum of diseases in a wide range of hosts, including man, other mammals, and birds. Upper respiratory and genital diseases are common clinical problems caused by *Chlamydiaceae*. Very little is known about chlamydial infections in dogs. Few clinical reports on natural disease in dogs describe mainly conjunctival and upper respiratory signs, and the role of *Chlamydiaceae *in genital disease is unclear. The present study aimed at studying the prevalence of *Chlamydiaceae *in healthy dogs and in dogs with genital or upper respiratory disease, including conjunctivitis.

**Methods:**

A real-time polymerase chain reaction (PCR) for *Chlamydiaceae *was used to detect any chlamydial species within this family. Swab samples from the conjunctiva and the mucosal membranes of the oropharynx, rectum and genital tract were taken from 79 dogs: 27 clinically healthy dogs, 25 dogs with clinical signs from the genital tract and 28 dogs with conjunctivitis. There were 52 female and 27 male dogs. From 7 of the male dogs, additional semen samples were analysed.

**Results:**

No *Chlamydiaceae *were detected from any dog.

**Conclusions:**

Although the number of dogs that was included is limited, the results suggest that cases of *Chlamydiaceae *in dogs probably are related to infection from other species, and that dogs in general do not harbour *Chlamydiaceae*. Bacteria belonging to the family *Chlamydiaceae *do not seem to be of major importance for genital or ocular disease in Swedish dogs.

## Background

Chlamydiae are obligately intracellular bacteria belonging to the bacterial order *Chlamydiales*. The *Chlamydiales *comprises four families: *Chlamydiaceae*, *Parachlamydiaceae*, *Simkaniaceae*, and *Waddliaceae*. The family *Chlamydiaceae *contains two genera: *Chlamydia *and *Chlamydophila *[1]. Bacteria belonging to the family *Chlamydiaceae *cause a broad spectrum of diseases in a wide range of hosts, including man, other mammals, and birds. Reproductive disturbances are common, among them salpingitis in man (caused by *Chlamydia trachomatis*), genitourinary disease in koalas (caused mainly by *Chlamydophila pecorum*) and abortions in sheep and cattle (caused mainly by *Chlamydophila abortus*) [2,3]. In cats there is also circumstantial evidence for a role of *Chlamydiaceae *in reproductive disturbances, and chlamydiae have been isolated from the vagina of cats, e.g. [4]. Bacteria belonging to the family *Chlamydiaceae *are also common ocular pathogens, for instance in humans *(C. trachomatis)*, koalas *(Cp. pecorum) *and cats (*Chlamydophila felis*). Avian chlamydiosis, caused by *Chlamydophila psittaci*, occurs worldwide and *Cp. psittaci *has been detected from a wide variety of both wild and domesticated birds. Avian chlamydiosis is the most important animal chlamydiosis transmissible to man (causing respiratory disease; ornithosis or psittacosis), but other chlamydiae, such as *Cp. abortus *and *Cp. felis*, also have a zoonotic potential [3], although *Cp. felis *infection in humans hitherto has been verified in only one, HIV-positive, case [5].

Very little is known regarding the potential of *Chlamydiaceae *to cause genital or upper respiratory disease including ocular disease in dogs. Detection of *Chlamydiaceae *requires special cultivation techniques, or analysis with polymerase chain reaction (PCR), and therefore these bacteria are seldom searched for in clinical cases of genital disease or reproductive disturbances. Other aetiological agents, such as canine herpes virus or extracellular aerobic bacteria, that are known to have the capacity of causing genital disease and reproductive problems in dogs, are searched for more frequently. Many cases of conjunctivitis are also treated on an empirical basis, without attempts to make an etiological diagnosis, and if a causative agent is searched for, this is rarely chlamydiae.

The aim of the present study was to study if members of the family *Chlamydiaceae *can be detected on the mucosa of healthy dogs, from dogs with reproductive disturbances or genital disease, or from dogs with upper respiratory signs including conjunctivitis. A real-time PCR that detects all members of the family *Chlamydiaceae *was used. No *Chlamydiaceae *was detected from any of the 4-5 sampling sites from the 79 dogs in the present study.

## Methods

### Ethical approval

The study was approved by the Uppsala ethical committee for animal research (C 310/05) and by the Swedish animal welfare agency (2006-0047). All dog owners gave their informed written consent.

### Dogs

79 privately own dogs of 41 different breeds, five mixed breeds and 5 with unknown breed, were included in the study. There were 52 females and 27 males. They were categorized as clinically healthy (N = 27), with clinical symptoms from the genital tract (N = 25), or from the upper respiratory tract, including ocular signs (N = 28). One bitch was sampled twice, first with cystic endometrial hyperplasia (CEH)/endometritis and 2 years later with conjunctivitis.

Signs from the genital tract included CEH/endometritis or vaginal discharge (N = 12), pyometra (N = 6), vulvar lesions (N = 1), poor semen quality (N = 1), failure to conceive (N = 1), prostatic disease (N = 1), aberrant oestrous cycles (N = 2) and balanoposthitis (N = 1). Upper respiratory signs included conjunctivitis (N = 20), conjunctivitis and cough or sneezing (N = 4), and only cough or sneezing (N = 4). The dogs were sampled with cotton tipped swabs without any transport or culture medium. From each dog, four samples were taken: from the conjunctiva, oropharynx, rectum and genital tract (vagina or preputium) by rolling swabs against the mucosa. From male dogs, an additional fifth swab was dipped in semen samples, when such a sample was available (N = 7).

### Real-time PCR analysis

At the laboratory, DNA was extracted from the swabs for PCR analysis according to the protocol by Sachse and Hotzel [6]. The samples were analysed using a real-time PCR originally developed by Everett et al. [2], which targets the 23 S ribosomal DNA and detects all members of the family *Chlamydiaceae*. We have modified this real-time PCR and developed an internal control to monitor any inhibition from the sample material [7].

## Results and discussion

Seventeen of the bitches (22%) were in heat and seventeen were in metoestrus. Of the 79 dogs, 27 (34%) were stud dogs or breeding bitches. For 24 dogs (30%), previous reproductive disturbances or genital disease was reported, and 26 dogs (33%) had previously had conjunctivitis. No *Chlamydiaceae *were detected in any sample.

The present study aimed at studying if healthy dogs or dogs with genital or upper respiratory including ocular disease harboured chlamydiae on their mucosa. In chronically infected ewes, the periovulatory period has been described to be optimal for detecting chlamydiae [8]. To increase the possibility of detecting chlamydiae in possibly infected bitches, several bitches (22%) were sampled in heat. Despite this, *Chlamydiaceae *were not detected from any of the samples.

Few seroepidemiological studies on chlamydiae in dogs have been performed. In a Japanese study, using a complement fixation test, 9.5% of dogs were seropositive for *Cp. psittaci*, which was an even higher number than for the cats in the same study [9]. There was also a large variation in seropositivity between different dog populations, from 0.9 to 17.4 per cent, with the highest seroprevalence in a colony of experimental animals. In a German study, using an enzyme-linked immunosorbent assay (ELISA), 20% of the dogs were seropositive for *Cp. psittaci*: 19.8% in a population of healthy dogs (N = 1028) and 26% of dogs with various clinical signs (N = 99) [10]. These results would indicate that infection with chlamydiae is not unusual in dogs. However, reports on naturally occurring chlamydial disease in dogs are scarce. Conjunctivitis caused by *Cp. abortus *has been described [11]. The source of infection could not be traced, but as the dog was living on the countryside, there were many possible infection routes directly and indirectly from sheep [11]. Coughing, dyspnoea, oculonasal discharge, pyrexia and anorexia caused by *Cp. psittaci *have been described: two out of three dogs in a household were infected, probably from a cockatiel [12]. The humans also developed severe clinical signs [12]. *Cp. psittaci *was also detected in a sample from a dog from a colony of dogs experiencing recurrent periods of dyspnoea, keratoconjunctivitis and small litter sizes and that had been kept together with a parrot and two canary birds [13]. Humans from the same household also had recurrent respiratory disease [13]. From one clinical sample from a dog with unknown clinical signs, *Cp. caviae *has been detected, a common chlamydial species in rabbits and guinea pigs, and from samples from 4 dogs with conjunctivitis, *Cp. felis *was detected [14].

A causative relationship between chlamydial infection and genital tract disease has not been reported in dogs, although small litter sizes were reported in a colony of dogs in which *Cp. psittaci *was detected in a sample from one of the dogs [13]. In an experimental setting, inflammatory changes in the prostatic gland have been induced by injecting *C. trachomatis *directly into the prostatic gland [15]. The dogs ceased to excrete chlamydiae after 3-16 days and no chlamydiae could be grown in tissue cultures, suggesting that the infection may have been self-limiting. No case of naturally occurring prostatic disease due to chlamydiae in dogs has been described, and no *Chlamydiaceae *were detected from the dog with prostatic disease in the present study. Although it has been suggested that the dog might be a model for chlamydial prostatitis [15] it does not seem likely that chlamydiae are natural causes of canine prostatic disease. Based on the scarcity of clinical reports, and the lack of naturally occurring chlamydiae on the canine mucosa found in the present study, chlamydial infection is likely an uncommon cause of reproductive disturbances in the canine species

Other clinical signs experienced in dogs infected with chlamydiae include hepatitis after experimental infection [16], and a suspicion of septic polyarthritis, although chlamydiae could not be cultured [17]. The organism could, however, be isolated from another dog with pyrexia and shifting leg lameness [18]. Chlamydiae have been isolated from pleural effusions of a dog referred to a clinic for pyrexia and shifting leg lameness. The chlamydiae were classified as "turkey strain" of *Cp. psittaci*, and it was speculated that the dog had been infected by an avian host [18].

Contact with another animal species infected with chlamydiae is thus almost always present when clinical disease caused by chlamydiae is present in dogs. Infection with chlamydiae is a potential differential etiological diagnosis in cases of especially upper respiratory disease in dogs with bird contact, and in cases of conjunctivitis after contact with cats (and maybe guinea pigs) with clinical chlamydiosis.

No case of suspected transmission of chlamydial infection between dogs has been reported. Chronic infection in dogs has been suggested, due to the identification of *Cp. pneumoniae *by PCR and immunohistochemistry from atherosclerotic lesions of aortas, coronary and splenic arteries of dogs [19]. As no chlamydiae were detected in the control animals, it was suggested that *Cp. pneumoniae *may play an important role in the development of canine atherosclerotic lesions [19]. Such changes are, however, uncommon in dogs. *Cp. pneumoniae *was neither detected in samples from dogs with idiopathic pericardial effusions [20], nor in samples from dogs with idiopathic lymphoplasmacytic rhinitis [21]. It has also been suggested that dogs may be potential reservoirs for *Cp. psittaci*, due to the persistent nature of the infection in a colony of dogs [13].

In Sweden it is required by law to report cases of ornithosis in humans to government authorities. The number of cases has decreased the last decennium, and since 2003 less than ten cases per year are reported http://www.smittskyddsinstitutet.se. The major source of infection is thought to be psittacine and other birds. Psittacosis in birds is also notifiable in Sweden, the number of cases being less than ten per year http://www.sjv.se. In Swedish dairy cows, *Cp. abortus *is absent or the prevalence is very low whereas *Cp. pecorum *was identified by PCR from vaginal swabs from 2/12 animals (17%) [22]. The prevalence of *Cp. psittaci *in faeces of passerine birds in Sweden is relatively low, around 3% (9/312, [23]). On the other hand, *Chlamydiaceae *is, in Sweden as in many other countries, commonly detected in samples from cats with conjunctivitis [24]. The risk for a dog to catch an infection with chlamydiae from sheep, cattle, or birds in Sweden is therefore rather low, whereas the risk of getting infected by a cat may be higher, although no such case was found in the present study.

## Conclusions

No *Chlamydiaceae *were detected from dogs in the present study. Although the number of dogs was limited, the results suggest that single cases of *Chlamydiaceae *in dogs are probably related to infection from other species, and that dogs in general do not harbour *Chlamydiaceae*. The frequency with which chlamydial infections can be diagnosed in dogs within a certain area can thus be expected to be dependent on the prevalence in other animal species, and the frequency of contact between dogs and these infected animals. Bacteria belonging to the family *Chlamydiaceae *do not seem to be of major importance for ocular or genital disease in Swedish dogs. Chlamydial infection could however be considered a potential differential diagnosis in dogs with upper respiratory disease and bird contacts, or with conjunctivitis and contact with cats.

## Competing interests

The authors declare that they have no competing interests.

## Authors' contributions

BSH designed and coordinated the study and had the major responsibility for writing and finalising the manuscript. SH, CLF and BSH collected the samples and GB was responsible for the PCR analysis. All authors participated actively in the writing of the manuscript, and all read and approved the final manuscript.
